# Soybean Embryonic Axis Transformation: Combining Biolistic and *Agrobacterium*-Mediated Protocols to Overcome Typical Complications of *In Vitro* Plant Regeneration

**DOI:** 10.3389/fpls.2020.01228

**Published:** 2020-08-12

**Authors:** Bruno Paes de Melo, Isabela Tristan Lourenço-Tessutti, Carolina Vianna Morgante, Naiara Cordeiro Santos, Luanna Bezerra Pinheiro, Camila Barrozo de Jesus Lins, Maria Cristina Matar Silva, Leonardo Lima Pepino Macedo, Elizabeth Pacheco Batista Fontes, Maria Fatima Grossi-de-Sa

**Affiliations:** ^1^ Biochemistry and Molecular Biology Department, Universidade Federal de Viçosa (UFV), Viçosa, Brazil; ^2^ Embrapa Genetic Resources and Biotechnology, Brasilia, Brazil; ^3^ National Institute of Science and Technology in Plant-Pest Interactions (INCTIPP), BIOAGRO, Viçosa, Brazil; ^4^ National Institute of Science and Technology, INCT PlantStress Biotech, EMBRAPA, Brasilia, Brazil; ^5^ Genomic Sciences and Biotechnology PPG, Universidade Católica de Brasília (UCB), Brasilia, Brazil

**Keywords:** *Glycine max*, genetic transformation, *Agrobacterium-*mediated transformation, particle bombardment, high-efficiency plant transformation, embryonic axis

## Abstract

The first successful attempt to generate genetically modified plants expressing a transgene was preformed via T-DNA-based gene transfer employing *Agrobacterium tumefaciens-*mediated genetic transformation. Limitations over infectivity and *in vitro* tissue culture led to the development of other DNA delivery systems, such as the biolistic method. Herein, we developed a new one-step protocol for transgenic soybean recovery by combining the two different transformation methods. This protocol comprises the following steps: agrobacterial preparation, seed sterilization, soybean embryo excision, shoot-cell injury by tungsten-microparticle bombardment, *A. tumefaciens*-mediated transformation, embryo co-cultivation *in vitro*, and selection of transgenic plants. This protocol can be completed in approximately 30–40 weeks. The average efficiency of producing transgenic soybean germlines using this protocol was 9.84%, similar to other previously described protocols. However, we introduced a more cost-effective, more straightforward and shorter methodology for transgenic plant recovery, which allows co-cultivation and plant regeneration in a single step, decreasing the chances of contamination and making the manipulation easier. Finally, as a hallmark, our protocol does not generate plant chimeras, in contrast to traditional plant regeneration protocols applied in other *Agrobacterium*-mediated transformation methods. Therefore, this new approach of plant transformation is applicable for studies of gene function and the production of transgenic cultivars carrying different traits for precision-breeding programs.

## Introduction

Genetic transformation is an essential key technique of genetic engineering tool kits. Modification of the genome allows us to discover the functions of genes and, consequently, the cellular processes under their control, raising many possibilities for biotechnological intervention and bioengineering. In plant science, genetic transformation has become a basic tool for precision genetic breeding, which allows specific characteristics to be directly encoded in the plant genome by foreign DNA delivery insertion or genome editing techniques.

Plant genetic transformation workflows are not trivial and their success depends not only on exogenous DNA insertion into the host genome but also on the regeneration of a whole-new functional and reproductive plant. Therefore, several studies have been conducted to enhance plant transformation and regeneration capacities and to develop easier protocols for execution, avoiding ordinary problems associated with *in vitro* tissue culture, the main limiting factor for precision genetic engineering in plant breeding (Rech et al., 2008).

There are two main methods of plant transformation according to the DNA-delivery system: a) particle bombardment, also called biolistic delivery (McCabe et al., 1988), and b) *Agrobacterium-*mediated transformation (Hinchee et al., 1988; Lee et al., 2009). The *Agrobacterium*-mediated method is still the most commonly used. *Agrobacterium ssp.* are plant-pathogenic bacteria that infiltrate plant cells by wounds and are capable of transferring and integrating T-DNA in plant-host genomes (Gelvin, 2000). Currently, optimized *Agrobacterium*-mediated protocols are successfully applied to generate transgenic plants; almost 85% of all species of transgenic plants have been generated by this method (Wu and Zhao, 2017). Perhaps it is suitable only for a few plant species, and the success of T-DNA transfer depends on several variable factors, such as bacterial virulence or the type of explant, which, combined with plant-explant recalcitrance and plant regeneration capacity, directly affect the success of plant transformation protocols.

The *Agrobacterium*-mediated method is highly reproducible, simple to operate, inexpensive and, mainly, allows one or a few insertions of exogenous DNA fragments in the host genome, which are its main advantages over the biolistic method (McCabe et al., 1988; Rech et al., 2008; Li et al., 2017). In general, several plant tissues can be used as explants for this transformation workflow, such as leaf nodes, epicotyls, hypocotyls, immature embryo axillary buds and cotyledonary nodes (Li et al., 2017), and the elementary steps of plant transformation are shared by many protocols. However, the steps of tissue cultivation and plant regeneration change completely according to the characteristics of each explant, and the choice of the explant type to be used should consider its regeneration capacity.

In soybean, cotyledonary nodes are often selected as the main explant type. They display a simple and efficient regeneration process (Kim et al., 1990; Sato et al., 1993; Li et al., 2017), and the transformation workflow is performed as follow: isolation of sterile explants, infection, cocultivation, shoot induction, root induction and, finally, seedling acclimation (Li et al., 2017). Normally, to ensure plant regeneration, phytohormones are applied in different combinations into plant culture media (co-cultivation medium (CCM), shoot elongation medium (SEM) and rooting medium (RM)) to promote shoot elongation followed by rooting. The most useful phytohormones are auxins, cytokinins, and gibberellins (Li et al., 2017), as they affect cell growth, tissue development, and plant regeneration. Auxin promotes cell elongation and plant growth, and cytokinins trigger cell division (Skoog and Miller, 1957; Normanly, 1997). When combined, they can induce callus formation or promote shoot elongation. On the other hand, a combination of auxin and gibberellin can stimulate root development (Nishijima, 2003; Thomas and Sun, 2004; Yamauchi et al., 2007; Gao et al., 2017).

The main source of troubles in plant transformation is the tissue culture step. In *Agrobacterium-*mediated protocols, the co-cultivation of bacteria and plant explants triggers defensive pathways in plants, culminating in the production of reactive oxygen species (ROS). ROS accumulation leads to tissue browning and necrosis, which limit the regenerative process (Li et al., 2017). To reduce tissue browning and enhance regeneration, CCM is often supplemented with antioxidants such as dithiothreitol (DTT), L-cysteine and PVPP (polyvinylpolypyrrolidone; Dutta Gupta, 2010; Li et al., 2017). Furthermore, the continuous manipulation and medium exchanges required during the process of plant regeneration are the sources of contamination with fungi and bacteria, decreasing the efficiency of the process.

These *Agrobacterium* transformation-derived troubles are partially overcome by biolistic protocols, in which exogenous DNA is directly bombarded against plant tissue and delivered to plant cells (Liu and Godwin, 2012). However, the biolistic method requires all the steps of tissue culture, and DNA bombardment frequently leads to undesirable multiple insertions. Additionally, it does not allow the transference of large DNA fragments and displays lower transformation efficiency than *Agrobacterium*-mediated methods (Li et al., 2017).

Rech et al. (2008) described a DNA bombardment-derived method for soybean, cotton and common bean transformation in which the embryonic axis is isolated from mature seeds and used as explants. The protocol avoids excessive tissue manipulation and explores the enhanced regenerative capacity of shoot meristematic cells. The limitation of this protocol is mainly in the selection of putative transgenic plants. Normally, selectable marker genes are widely used to ensure the regeneration of only transformed cells in a selective culture medium supplied with antibiotics or herbicides. Using embryonic axis as explants, the shoot meristematic cells are not in contact with the selective agent once just the embryo radicle is immersed into the selective culture medium. The embryos are nourished by the radicle-shoot axis and molecules that are systemically translocated and capable of accumulating on shoot cells are suitable for use as selective agents. To date, only the Imazapyr herbicide can be used for this purpose. Imazapyr is an imidazolinone-based herbicide that inhibits the activity of acetohydroxyacid synthase, which disrupts the biosynthesis of the amino acids leucine, isoleucine, and valine (Shaner et al., 1984). Plants carrying the mutated *Arabidopsis thaliana ahas* gene display specific imidazolinone resistance and are suitable for selection with imazapyr during the transformation workflow.

Herein, we provide a complete protocol for soybean embryonic axis transformation mediated by *A. tumefaciens*. It has already been described a combination of particle-bombardment and *A. tumefaciens*-mediated protocols using embriogenic callus from half-seeds as plant-explant (Droste et al., 2000). Despite presenting a new method for soybean transformation, the work reported low-efficiency compared with other methods. In addition, the choice of callus as plant explant does not eliminate ordinary complications arising from tissue culture steps. Recently, Pareddy et al. (2020) described a method of *Agrobacterium*-mediated transformation with approximately 19% of transformation efficiency, superior to other already described. The new methodology takes as explant the half-imbibed seeds and explores the competence of shoot cells in the embryonic axis for genetic transformation and regeneration by *in vitro* organogenesis. Mechanical removal of radicle system enhances the *Agrobacteria* infectivity and contributes to high reported efficiency. However, the plant regeneration demands *in vitro* organogenesis and the plant recovery workflow is superior to 15 weeks. For shoot induction, elongation and rooting steps, continuous media-changes and supplementation with several phytohormones and growth regulators, such as BAP, timentin, IAA, zeatin, and GA_3_, are required. In spite of the high efficiency of transgene-integration, these protocol’s features make it long, expensive and do not overcome ordinary complications of *in vitro* plant regeneration, which comprises the limiting step on soybean transformation workflow.

Our protocol explores the main advantages of each soybean transformation system to overcome typical issues on plant regeneration, making it easily reproductive into obtaining transgenic fertile-lines in a direct and time- and cost-optimized way, with reasonable transformation efficiency. These advantages include i) the particle acceleration by biolistic systems for explant wounding, which is required for *Agrobacterium*-mediated plant infection; ii) the capacity of *A. tumefaciens* to transfer exogenous large foreign DNA as single or few copies in the host genome; and iii) the use of the embryonic axis as a plant explant. The shoot cells of the embryonic axis display high transformability and regeneration capacity and can be submitted to a one-step transformation, co-cultivation, and regeneration workflow, avoiding excessive manipulation of explants, comprising the main advantage over other organogenesis-based methods. These combined features reduce the challenges in soybean transformation and enhance the efficiency of the process, making it more suitable for studying gene function and generating new engineered cultivars.

## Materials and Equipment

### General Reagents, Equipment, and Materials

- Sterile distilled and deionized water
- Absolute ethanol
- Isopropanol
- Tweezers
- Glass sterile Petri dishes (135 mm diameter)
- Scalpel (n.10)

### Bacterial Culture

- Luria-Bertani (LB) medium
- Bacteriological agar
- Gentamicin
- Rifampicin
- Kanamycin

### Biological Material

- Mature seeds of soybean Williams 82
- *tumefaciens* GV3101 with the desired construct

### Embryo Transformation and Plant Tissue Culture

- Sodium hypochlorite (2.5% v/v)
- Sterile Whatman paper
- Glycerol
- B5 basal plant medium (Gamborg)
- MES buffer (2-N-morpholinoethanosulfonic acid)
- Sucrose
- DTT (dithiothreitol)
- BAP (6-benzylaminopurine)
- Acetosyringone (4′-hydroxy-3′,5′-dimethoxyacetophenone)
- GA_3_ (gibberellin analog)
- L-cysteine
- Sodium thiosulfate
- MS (Murashige and Skoog) plant medium
- Activated charcoal
- Imazapyr (2-(4,5-dihydro-4-methylethyl)-5-oxo-1H-imidazol-2-yl)-3-pyridinecarboxylic acid)
- Tungsten microparticles
- Carrier membrane (50 µm thickness and 24 mm diameter)
- Rupture disks (250 µm thickness and 13.2 mm diameter)
- Petri dishes (5 cm diameter)
- Magenta plant tissue culture boxes

### Equipment

- Biological laminar flow chamber
- Autoclave
- Shaker incubator
- Centrifuge
- (Bio)spectrophotometer
- Helium pressure-driven microparticle acceleration system
- Ultrasonic bath
- Standard plant tissue culture room and greenhouse

### Reagent Set-Up

**Acetosyringone Stock Solution**: Dissolve 1.96 g of acetosyringone (3,5- dimethoxy-4-hydroxy-acetophenone) in 100 ml of ethanol (100%) to obtain a final concentration of 100 mM and store in a refrigerator at 4°C.

**BAP Stock Solution**: Dissolve 50 mg of 6-BAP in 1 N NaOH, complete the volume to 10 ml with distilled water, sterilize by autoclaving along with the culture medium. Store at -20°C for up to 6 months.

**GA_3_ Stock Solution**: Add 100 mg of gibberellic acid 3 to 100 ml of distilled water and stir until dissolved to make a solution with a final concentration of 1 mg/ml (1000 ppm). It is best to use this solution fresh. However, a stock solution can be stored in the dark at -20°C for up to 6 months. Sterilize the solution by filtration (0.22 µm)

**Imazapyr Stock Solution**: Prepare a stock solution to a 1 mM final concentration. Dissolve 13.17 mg of imazapyr in 50 ml of distilled and deionized sterile water. Using a microfilter (0.22 µm), sterilize the solution and stock 1 ml aliquots in aluminum-protected tubes at -20°C for up to 1 month.

**Tungsten Particle Pre-Preparation**: Separate 60 mg of tungsten particles in a sterile tube and add 1 ml of 70% ethanol (v/v). Homogenize the suspension by vortexing for 15 min. Centrifuge at 3,000 g—5 min. Remove the ethanol with a pipette without disrupting the pellet. Add 1 ml of sterile distilled and deionized water, mix very well by vortexing, centrifuge as described and repeat the cleaning steps 3 times. At the last washing, remove the water and resuspend the microparticles with 1 ml of sterile 50% glycerol (v/v). Store the particles at -20°C.

## Methods

**Figure 1:**
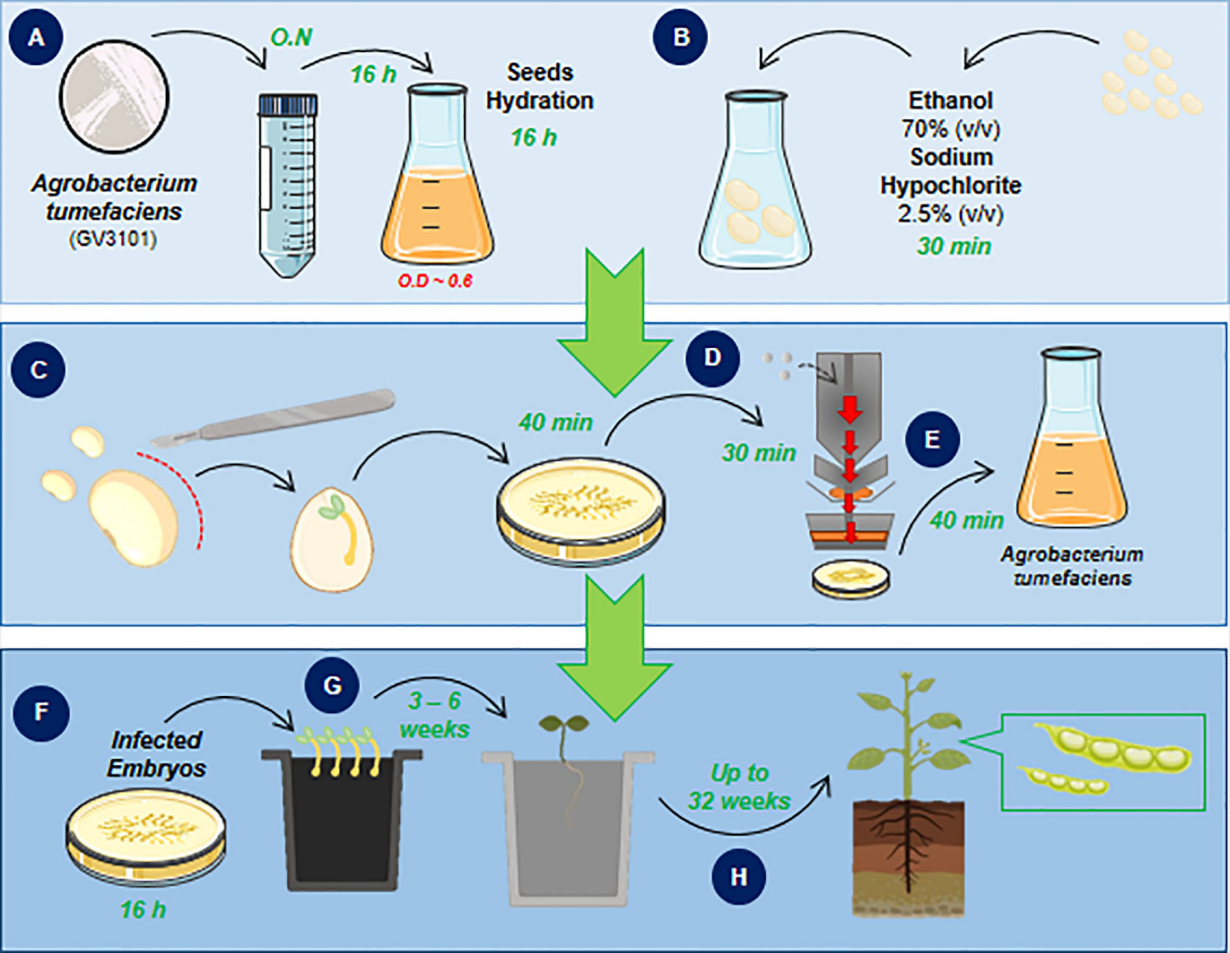
Biolistic and *Agrobacterium-*mediated soybean embryonic axis transformation workflow. The workflow of the proposed protocol in this study consists of a one-step soybean embryo infection followed by plant regeneration to obtain transgenic soybean plants. The workflow is divided into three main parts that are followed day-by-day until the plant regeneration step, which requires up to 6 weeks until seedling acclimation. The protocol starts with isolation and bacterial culture **(A)** in parallel with seed sterilization and hydration **(B)**. The next step achieves the isolation of embryonic axis **(C)**, bombardment of shoot cells that will be transformed and regenerated into a reproductive plant, **(D)** and, finally, *A. tumefaciens* (GV3101 strain) infection **(E)**. After embryo infection, cocultivation occurs **(F)** and is immediately followed by plant regeneration **(G)** until the acclimation step and plant recovery **(H)**.

The following procedures introduce a one-step method for soybean embryonic axis transformation by *A. tumefaciens*. The workflow describes the process of embryo isolation, explant infection, plant-bacteria co-cultivation and putative transgenic plant regeneration and selection **(** **Figure 1** **)** to overcome issues and, consequently, low efficiency. All described steps were adjusted for transformation of 150 embryo axes.

### Day 1 ‖ Bacterial Preparation • Time: 30 min + 16 h

1. To prepare an *Agrobacterium* culture, pick up a single colony of *A. tumefaciens* GV3101 that had been previously transformed with the vector of interest to inoculate 5 ml of LB medium supplemented with 50 mg/L gentamicin, 100 mg/L rifampicin and 100 mg/L kanamycin (or a corresponding dosage of a plasmid-selective agent).
2. Incubate in shaker under agitation—180 rpm—28°C—overnight (ON).
3. After bacterial growth, dilute 100 µl of preinoculum into 100 ml of LB medium with the same concentrations of the selective agents. Incubate under the same conditions until the OD_600_ = 0.6 (~ 16 h). **▲CRITICAL** The OD_600_ accuracy at this point is very important to guarantee bacterial virulence. At this OD, the bacterium culture is in the log phase, and its metabolism is completely active. The culture can be stored in the refrigerator (4°C) until embryo incubation.

### Day 1 ‖ Plant Culture Medium • Time: 180 min

Initial notes:

- 100 ml of liquid CCM for each round of transformation/plasmid.
- 20 ml of solid CCM for each bombardment plate containing 30 embryos

P.S. 300 embryos (distributed on 10 plates) are normally bombarded at one round of transformation.

CCM contains 0.3 g/L B5 basal medium (Gamborg), 3.9 g/L MES (2-N-morpholinoethanosulfonic acid), 30 g/L sucrose and 154.2 mg/L DTT (dithiothreitol).

1. Separately dissolve all the components of CCM in distilled and deionized water.
2. After medium preparation, adjust the pH to 5.4.
3. Sterilize by autoclaving.
4. The liquid medium can be stored at 4°C for 1 week.

To prepare solid CCM, follow the protocol for preparation of liquid medium.

1. Supplement the liquid medium with 400 mg/L L-cysteine and 158 mg of sodium thiosulfate.
2. After pH adjustment, add 5 g/L bacteriological agar.
3. Sterilize by autoclaving.
4. Supplement the sterile medium with 835 µg/L BAP (6-benzylaminopurine), 40 mg/L acetosyringone and 0.25 mg/L GA_3_ (gibberellin analog), all filter-sterilized, and immediately distribute it in 5 cm (ϕ) plastic Petri dishes (approximately 20 ml medium/plate), forming a layer of 0.6–0.7 mm. **▲CRITICAL** Do not add the sterile hormones to hot medium. The ideal temperature for adding hormones to a culture medium is 55°C, preferably measured by an infrared thermometer. Add the acetosyringone only when resuspending the bacterial culture.

The development and root medium (DRM) contained 4.3 g/L MS (Murashige and Skoog) medium, 30 g/L sucrose, 1.0 mg/L BAP, and 1.0 g/L activated charcoal.

1. Take all the components of the DRM except the charcoal and dissolve them in distilled and deionized water.
2. Adjust the pH to 5.7.
3. After the pH adjustment, add 5.0 g/L bacteriological agar and activated charcoal. **▲CRITICAL** Do not shake the flask to mix the components. The charcoal is not soluble in water and mixing it by shaking spreads the particles; hence, they will stay heterogeneously distributed in the final medium.
4. Supplement DRM with a selective agent (herbicide) according to the literature-recommended dosage. **▲CRITICAL** If the selective marker gene is *bar*, which confers resistance to ammonium glufosinate-derived herbicides, the DRM should not be supplemented with ammonium glufosinate. Ammonium glufosinate is not a systemic herbicide and requires the contact between transformed cells and culture medium. During plant regeneration, apical meristem cells are not in contact with the culture medium; hence, non-transformed radicle cells will not be in contact with the herbicide (Zhang et al., 1999; Li et al., 2017). The selection of putatively transformed plants is performed after complete plant regeneration by herbicide pulverization in a greenhouse. However, if the selective marker gene is *ahas*, which confers imidazolinone resistance, the DRM should be supplemented with 600 nM imazapyr. Imazapyr is sensitive to light and heat. Keep aliquots in an amber flask until utilization and add them to cold medium (55°C).

### Day 1 ‖ Seed Sterilization • Time: 30 min + 16 h

**(!) CAUTION** All the following steps should be performed in a laminar flow chamber previously cleaned with sodium hypochlorite (2.5% v/v) and ethanol (70% v/v) and exposed to UV radiation for 20 min.

1. As only 50% of the embryonic axis is recovered for transformation during the isolation process, it is recommended to sterilize double the number of seeds. For example, if you start with 600 seeds, you will have approximately 300 embryonic axes viable for transformation. Distribute the seeds in two sterile 350 ml Erlenmeyer flasks. Immerse the seeds in 75% (v/v) ethanol for 2 min. The following steps are adjusted for embryonic axis isolation from 300 seeds and transformation of 150 embryo axes.
2. Remove the ethanol and immerse the seeds in commercial sodium hypochlorite (2,5% v/v) for 20 min, gently mixing every 5 min.
3. Remove the sodium hypochlorite and rinse the seeds 3 times with sterile water.
4. Immerse the seeds with sterile water leaving a 3 cm layer above them; seal the Erlenmeyer and store under dark for 24 h until embryo isolation. **▲CRITICAL:** Floating seeds should be discarded.

### Day 2 ‖ Preparation of Tungsten Microparticle-Carrying Membranes • Time: 30 min

**Figure 2:**
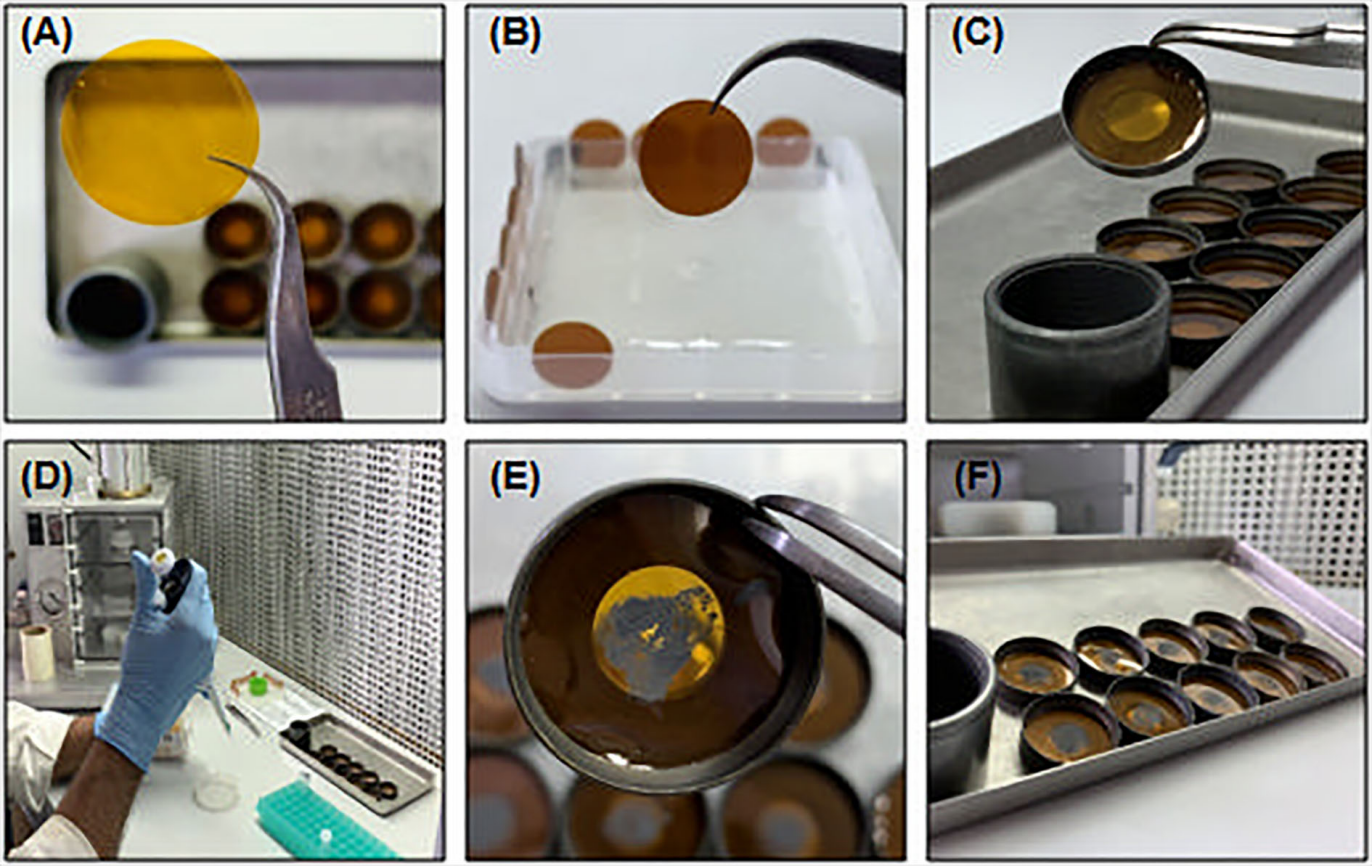
Tungsten-coated carrier membrane preparation. **(A)** Carrier membrane (24 mm diameter); **(B)** Rupture disk (13.2 mm diameter); **(C)** Metallic ring support of carrier membrane; **(D, E)** Distribution of prepared tungsten microparticles at carrier membrane surface; **(F)** Prepared tungsten-coated carrier membranes under hood to drying. All manipulation in tungsten-coated membranes’ preparing may be performed under pre-cleaned and UV-sterile hood.

The microparticle accelerator system demands two types of membranes: the carrier membrane (24 mm diameter, **Figure 2A**), where tungsten is deposited to be accelerated against plant tissue, and the rupture disk (13.2 mm diameter, **Figure 2B**), for sealing the helium at the high-pressure chamber in the particle accelerator.

The setup of this transformation requires a 1200 p.s.i. rupture pressure. If necessary, use more than one rupture disk to achieve the desired pressure, *e.g.*, 4 x 300-p.s.i. rupture disks.

The carrier membranes are attached to a metallic ring that should be stored in ethanol and flame-sterilized before use (**Figure 2C**).

Particle preparation:

- 1 carrier membrane for each round of bombardment. P.S. 150 embryo axes divided into 7–8 bombardment plates may be available at this step.
- 4 rupture (300 p.s.i.) disks for each round of bombardment.

1. Vortex the pre-prepared aliquot of tungsten microparticles 1 min and homogenize it very well.
2. Separate an aliquot of 100 µl in a sterile microtube.
3. Add 100 µl of sterile distilled and deionized water and homogenize gently.
4. Centrifuge at 3,000 g—30 s.
5. Carefully remove the supernatant with the help of a micropipette. Take care to not aspirate the particles at the bottom of the tube.
6. Add 300 µl of absolute ethanol and homogenize gently.
7. Centrifuge 30 s at 3,000 g.
8. Repeat steps 6 and 7 twice.
9. Carefully remove the supernatant and add 48 µl of absolute ethanol to the particles.
10. Sonicate the particles for 30 s at maximum intensity to assure that the tungsten particles are well separated, which provides a better distribution at the carrier membrane surface.
11. Attach the ethanol-humidified carrier membrane to the metallic ring. **▲CRITICAL** The membrane should be very well attached to the metallic ring, avoiding roughness and irregularities at the surface of the membrane. This step is important for the correct acceleration of tungsten particles and their homogeneous distribution at the embryo axis shoot meristem.
12. Using a micropipette, homogenize the particles and distribute 8 µl at the center of ethanol-humidified membranes **(** **Figures 2D, E** **)**. **▲CRITICAL** Do not distribute the particles on dry membranes. The ethanol at the surface of the carrier membrane ensures the homogeneous distribution of tungsten particles.
13. Distribute the metallic-coated membranes on a sterile petri dish and let them dry under a hood **(** **Figure 2F** **)**. The membranes are kept under a sterile atmosphere until utilization.

### Day 2 ‖ Embryonic Axis Excision and Shoot Exposure • Time: 180 min

**(!) CAUTION** All the following steps should be performed in a hood previously cleaned with sodium hypochlorite (2.5% v/v) and ethanol (70% v/v) and exposed to UV radiation for 20 min.

**Figure 3:**
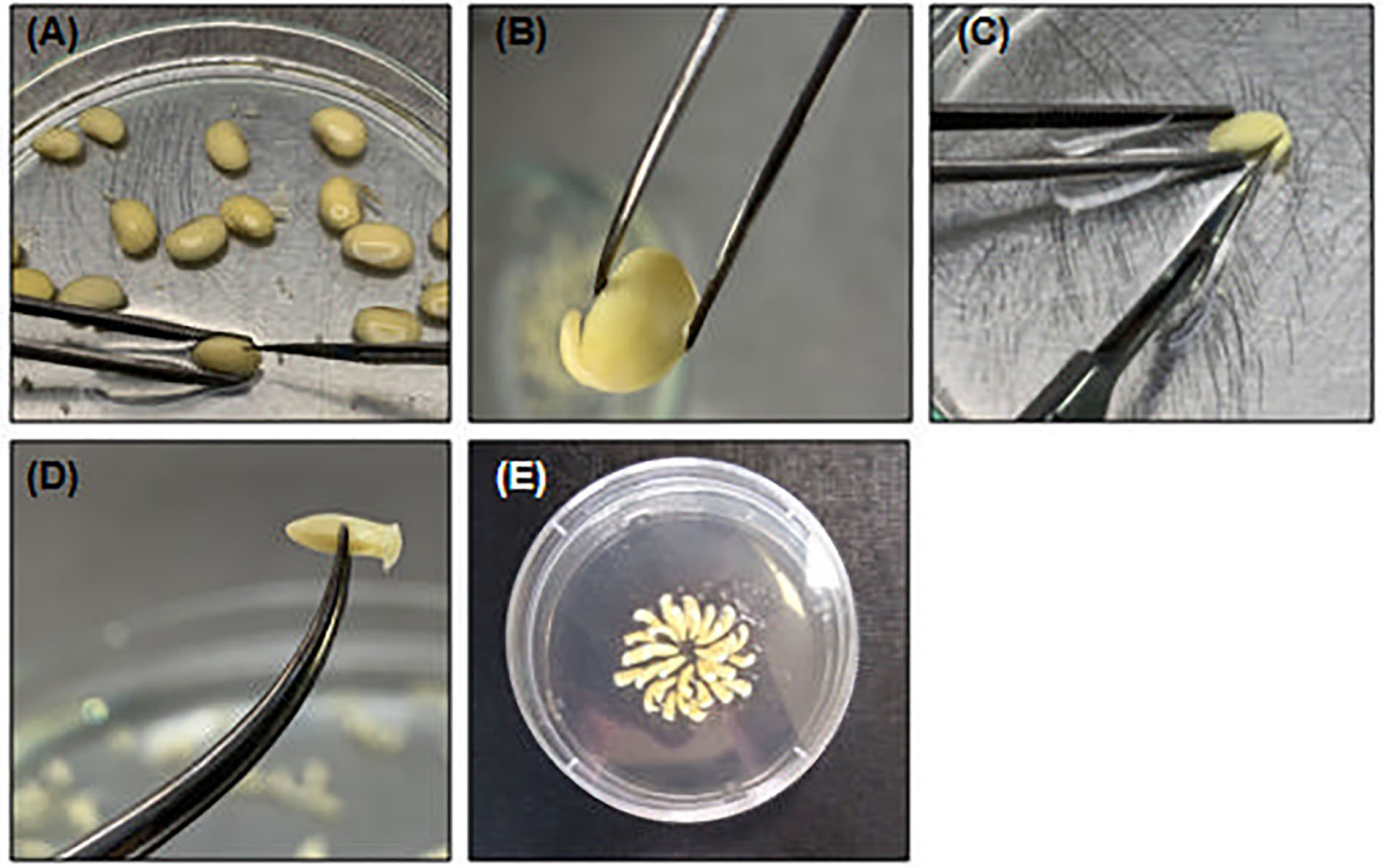
Embryonic axis excision and shoot exposing. **(A)** Pick one hydrated soybean seed and with help of a tweezes and a scalpel make a small section in the opposite of hilo; **(B)** Discard the embryo non-associated cotyledon and **(C)** carefully detach the embryo axis with scalpel. **(D)** Remove the primary leaves to expose shoot cells; **(E)** Organized embryo in CCM to bombardment.

1. Spread the hydrated seeds in a plate dish filled with fresh sterile water.
2. Pick up each seed individually with a flame-sterilized tweezers and make a longitudinal section opposite to the hilo **(** **Figure 3A** **)** with the help of a new and flame-sterilized scalpel (n. 10). **▲CRITICAL** To avoid embryo damage, do not insert the scalpel very deep.
3. Separate the half-cotyledon attached to the embryo and discard the other part. Turn the half-cotyledon with the abaxial surface in contact with the plate. With the help of the scalpel and tweezers, press the opposite side of the embryo until embryo detachment **(** **Figures 3B, C** **)**.
4. Remove the primordia to expose the shoot meristem cells **(** **Figure 3D** **)**.
5. With the tip of a scalpel, repeatedly move the tip of the embryo with friction to ensure the exposure of the shooting area.
6. Transfer the isolated embryo to a petri dish with sterile distilled and deionized water.

### Day 2 ‖ Preparation of Embryos for Bombardment • Time: 40 min

1. Organize the embryos at the CCM solid (petri dish 5 cm ϕ) for bombardment. Make a bisected circle with 15 overlapped embryos in each line **(** **Figure 3E** **)**. **▲CRITICAL** The embryonic axis should be positioned with the shoot meristem directed upward and lightly angled to enhance the penetration of tungsten particles at the particle distribution radius. It is also important to avoid the distribution of embryos into the “death zone”, where the incidence of microparticles is higher and the shock of tungsten particles to shoot cells is more intense. It can generate damage to the tissue, making it unviable for genetic transformation.
2. Close the Petri dishes, seal them with parafilm and keep the embryos in a sterile environment until bombardment. **▲CRITICAL** Avoid water-film formation after embryo axis preparation. The water accumulated at the surface of shooting cells acts as a block for microparticle penetration, thereby reducing the wounding ratio and the frequency of transformation.

### Day 2 ‖ Agrobacterium Preparation • Time: 50 min

1. Under the pre-cleaned hood, transfer the *A. tumefaciens* culture to sterile 50 ml tubes.
2. Centrifuge at 5,000 g—10 min.
3. Discard the supernatant, gently resuspend the pellet in 50 ml of CCM supplemented with acetosyringone, and transfer it to a sterile 100 ml Erlenmeyer flask.
4. Incubate the CCM-resuspended *Agrobacterium* in shaker under agitation—180 rpm—28°C—30 min. **▲CRITICAL** The incubation of CCM-resuspended bacteria is an important step to enhance bacterial virulence. The acetosyringone in CCM will activate virulence genes, which are required for T-DNA transfer during the embryo infection step.

### Day 2 ‖ Soybean Embryo Axis Bombardment • Time: 30 min

Follow straightly the manufacturer instructions to use the helium pressure-driven microparticle acceleration system. This protocol utilizes the following settings: the pressure of helium entering the high-pressure chamber—1,200 p.s.i; vacuum pressure under the bombardment chamber—25 mmHg.

**Figure 4:**
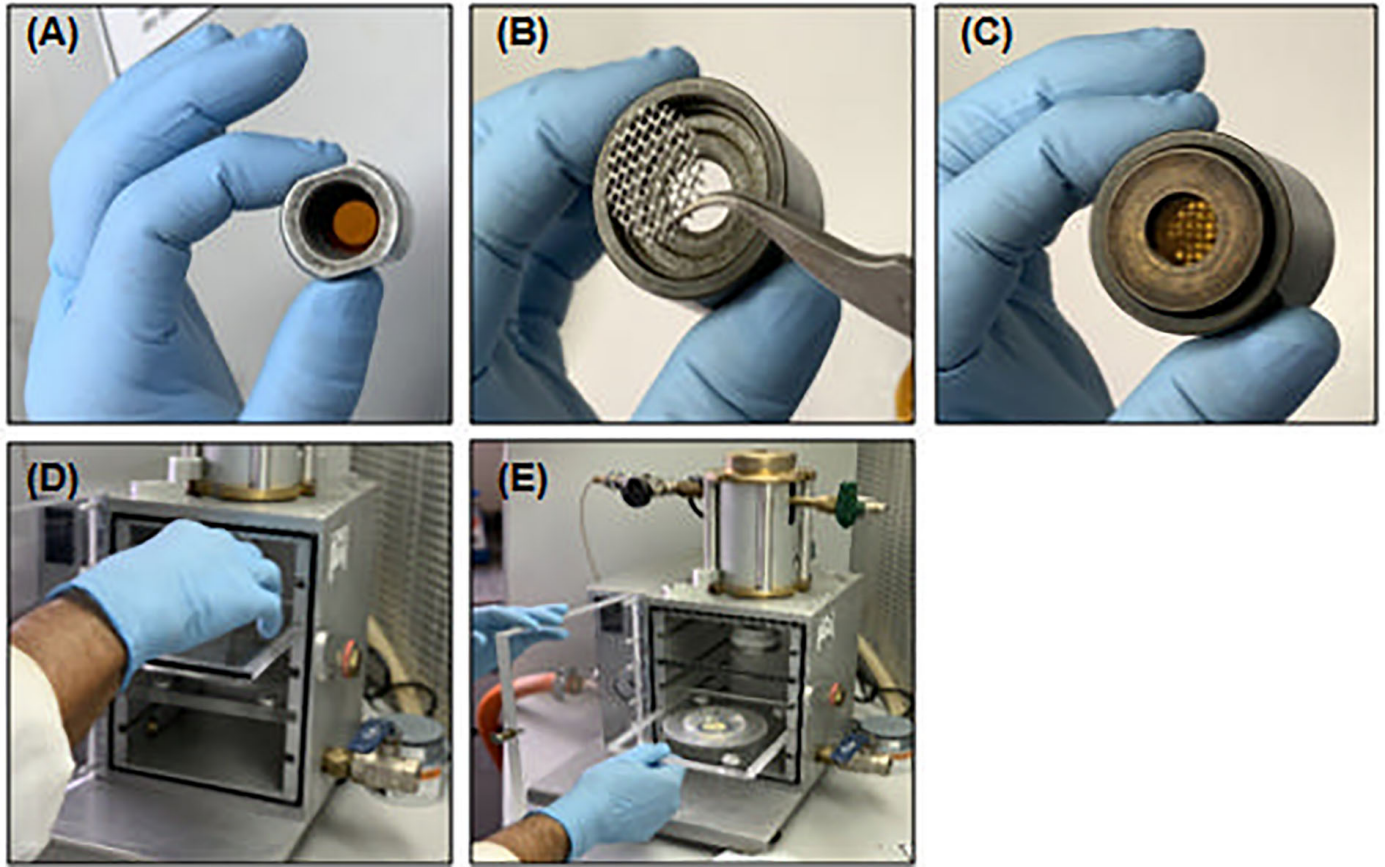
Soybean embryo axis bombardment. **(A)** Isopropanol-soaked rupture disks attached to the tip of helium pressure chamber; **(B)** Protective metallic net at the support of carrier membrane; **(C, D)** Carrier membrane attached to ring support at holder in bombardment chamber; **(E)** Embryonic axis at CCM plate into bombardment chamber.

1. Attach four isopropanol-soaked rupture disks at the tip of the helium trigger **(** **Figure 4A** **)**. **▲CRITICAL** Avoid air bubbles between the membranes to reach the correct pressurization of the helium chamber. Isopropanol promotes better adhesion of membranes and chamber sealing.
2. Place the metallic net protection at the support of the carrier membrane **(** **Figure 4B** **)**. The protective net avoids plastic particle spreading, which results from membrane rupture, under the soybean embryo axis.
3. Attach the tungsten-coated carrier membrane at the support and close it tightly **(** **Figures 4C, D** **)**.
4. Put the opened petri dish with the soybean embryo axis at the center of the bombardment chamber **(** **Figure 4E** **)** and close the hermetic door.
5. Activate the vacuum pump to set the pressure at 25 mmHg in the bombardment chamber. Close the airflow valve and open the helium flow until the helium high-pressure chamber is filled. **▲CRITICAL** The time it takes for the high-pressure chamber to fill is 3–5 s. Excessive helium pressure disrupts the protective disks and triggers the energized needle, liberating the helium pressure under the embryo axis prematurely.
6. Trigger the energized needle. The helium pressure will trigger the needle towards the carrier membranes, and the mechanical shock between gas and microparticles will spread them over the shoot meristems at high speed, generating micro-wounds, which are required for *Agrobacterium* infiltration.
7. Release the pressure under the bombardment chamber and helium chamber, subsequently. Remove the embryo axis of the bombardment chamber and close the petri dish with parafilm until *Agrobacterium* incubation.

### Day 2 ‖ *Agrobacterium Tumefaciens*-Mediated Transformation and Embryo Cocultivation • Time: 60 min + 16 h

**Figure 5:**
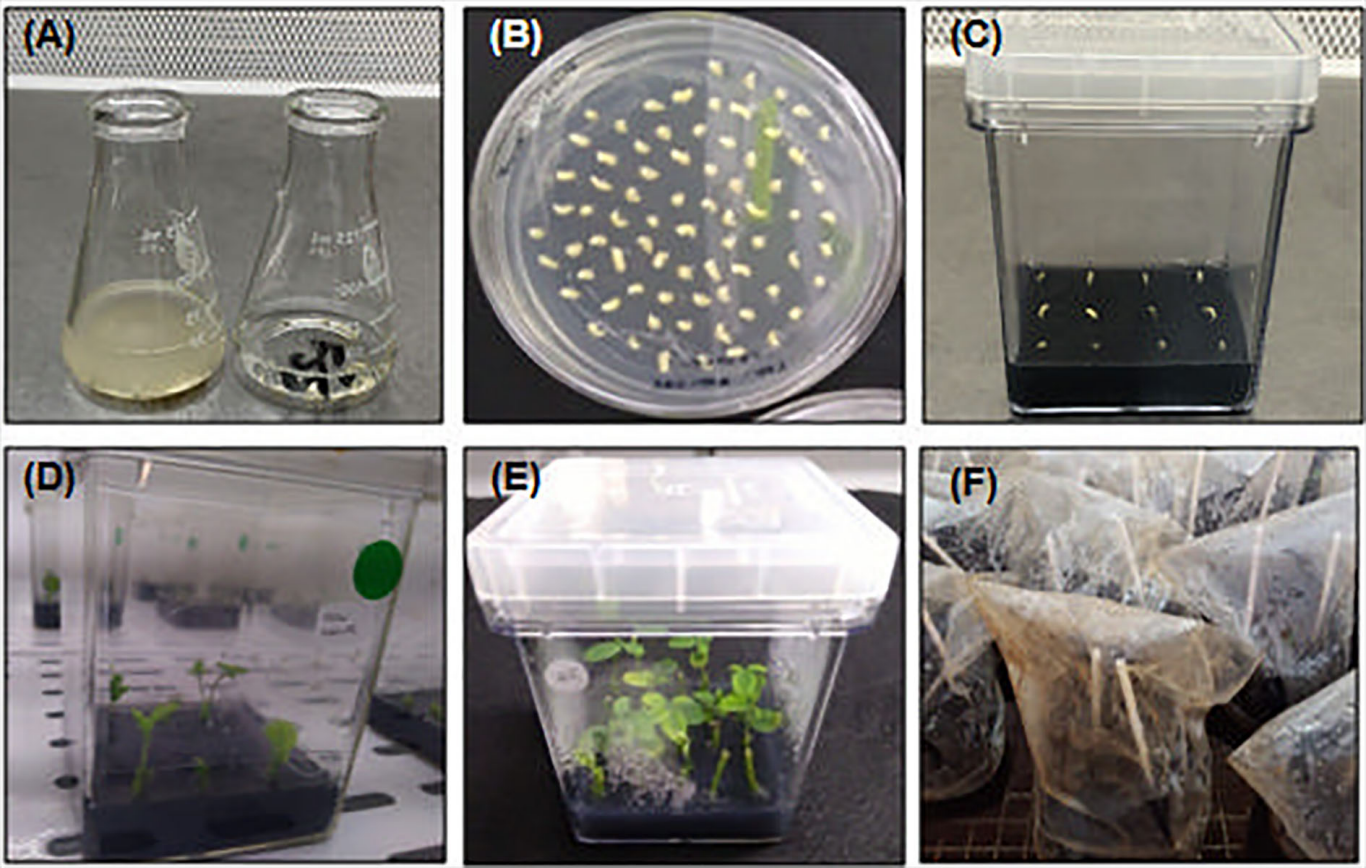
*Agrobacterium tumefaciens* infection, co-cultivation and plant regeneration. **(A)** Bombarded embryos in *Agrobacterium* suspension (left) and after washing (right), performed after 40 min of infection; **(B)** Infected embryos at CCM. The duration of co-cultivation step at CCM is 16 h; **(C)** Infected embryos at DRM after co-cultivation in CCM. The plant-regenerative process starts as from the transference of infected embryos to DRM. The *A. tumefaciens* is not inactivated by chemical treatment and the infection continues along with plant regeneration. At this point, plant surveying is essential to avoid contamination, which should be eliminated by transferring the plant to a new medium or by chemical treatment, if necessary; **(D, E)** Shoot- and root-regenerated seedlings after 3–6 weeks in DRM. **(F)** After complete seedlings’ regeneration, the plants can be acclimated in standard greenhouse protected with a plastic bag for 1 week and follow normal cycle of development until seeds recovery. All steps of plant characterization can be performed at this point.

1. Carefully remove the bombarded embryo axis from the CCM plate with the help of a flame-sterilized tweezers. To avoid embryo damage, do not squeeze tweezers tightly.
2. Immerse the embryos on CCM-resuspended bacteria **(** **Figure 5A** **)** and incubate the co-culture under shaking—120 rpm—28°C—40 min.
3. Remove the bacterial culture and wash the embryos with sterile distilled and deionized water 3 times.
4. Pour off the water and transfer the embryo axis to sterile Whatman filter paper to remove excess water **(** **Figure 5A** **)**.
5. Arrange the embryo axis in the CCM petri dish with the radicle completely immersed in the medium **(** **Figure 5B** **)**. The hormones and metabolites present in the medium will stimulate the development of shoot and root meristem and *Agrobacterium* infection.
6. Seal the plates with parafilm. Keep the infected embryos under darkness for 16 h until transference to DRM.

### Day 3 ‖ In Vitro Culture of Soybean Embryonic Axis • Time: 60 min + 3–6 weeks

1. Transfer the embryo axes to the DRM. **▲CRITICAL** The positioning of the embryo axes at the DRM should be well organized. Each magenta box containing the DRM has 12 embryo axes (4 × 3) distributed equidistantly **(** **Figure 5C** **)**.
2. Transfer the magenta boxes with infected embryos to an *in vitro* culture room—28°C—16 h photoperiod—50 µmol.m^-2^.s^-1^. After 3–6 weeks in the DRM, the putatively transformed shoot meristem cells develop into elongated shoots, and the radicles develop into roots **(** **Figures 5D, E** **)**.

**(!) CAUTION** The protocol of soybean embryonic axis transformation mediated by *A. tumefaciens* is a co-cultivation, shoot and root elongation one-step protocol. Therefore, the bacteria from the embryo axis are not removed or eliminated by antibiotics or any other chemical treatment. During the *in vitro* culture period, embryos should be surveyed daily. Excessive bacterial growth can be controlled by treating the embryo axes with cefotaxime solution (150 mg/L) after co-cultivation. Fungal and/or bacterial contamination sources can affect seedling development and decrease the efficiency of transformation, leading to a very low success ratio. During 3–6 weeks of shoot and root elongation, healthy embryos were transferred to another DRM magenta box if contamination spots appeared and contaminated embryos were eliminated.

**Figure 6:**
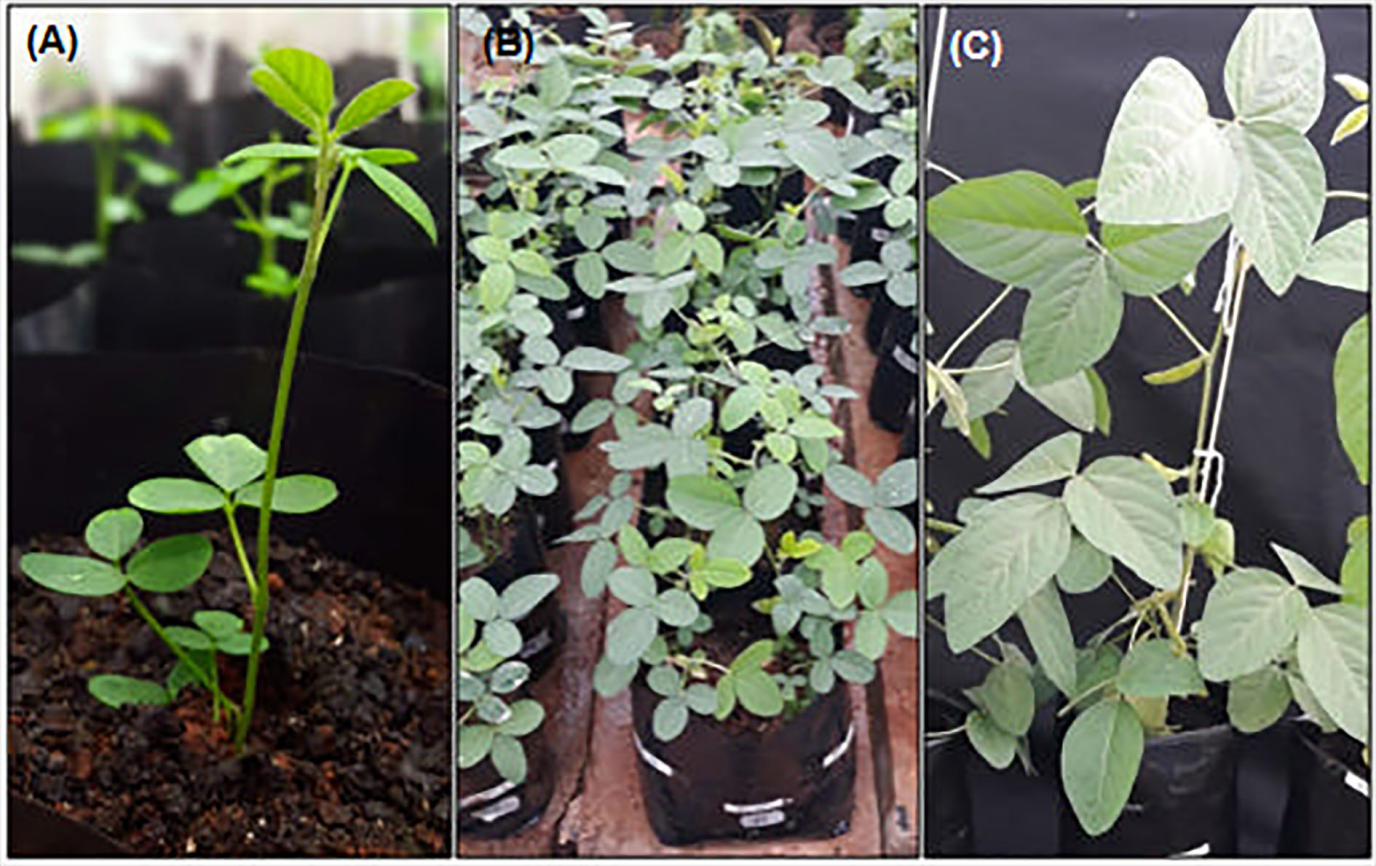
Regenerated plants under full development in greenhouse. **(A)** *In vitro* recovered seedling after DRM-regeneration and 1-week of plant acclimation. **(B)** Putative transgenic T0-matrixes in vegetative stage. **(C)** T1-recovered plants in reproductive stage.

### Up to 6 weeks ‖ *Greenhouse Plant Development* • Time: 24–32 weeks

1. Transfer the shoot- and root-developed plants to individual 1 dm^3^ (1.0 L) pots filled ¾ with fertilized soil:vermiculite (2:1).
2. Cover the plants with a transparent plastic bag supported on side straws and seal with a rubber band (**Figure 5F**).
3. Keep the plants in a greenhouse under normal development—27—30°C—14 h photoperiod and 75% relative humidity. **▲CRITICAL** The soil humidity should be checked daily. Take care with the water drops generated at the surface of the plastic bag during the evapotranspiration of seedlings returning to the soil.
4. After 1 week, remove the plastic bag and perform a careful watering of seedlings, avoiding soil erosion and root exposure by strong water flow (**Figure 6A**).

The acclimatized plants still under normal development in the same pot until seed recovery, normally complete at 30 weeks after acclimation (**Figures 6B, C**). During this period, standard procedures of plant care may be adopted to keep plants healthy and productive. The selection of putative transformants should be performed during this period. For ammonium glufosinate-mediated selection, prepare a 125 mg/L solution and, with the help of a swab, spread the solution in half of some leaves. Resistant plants can be adopted as putative transgenic plants.

The final diagnosis of transgenic plants can be performed by PCR or other analytical methods to detect the presence of the foreign gene and/or by methods to detect the protein. In our protocol, we performed an analysis of melting temperature (Tm) by qPCR. We compared the Tm of amplicons generated by a reaction performed with 50 ng of standard plasmid DNA (used to transform *A. tumefaciens*) and 50 ng of plant DNA as follows: 94°C for 3 min; 40 cycles of 94°C for 5 s and 56°C for 40 s, followed by a melting step with a resolution of 0.5°C.

## Anticipated Results

The newly described protocol was validated with five different series of soybean embryo axis transformations that employed distinct cassettes for transformation harboring different vector backbones and selective marker genes. In the presence of a selective agent in DRM, the regeneration ratio (rounds 1, 2, and 5—**Table 1**) was 31.16%. In its absence, the regeneration rate reached almost 86% (rounds 3 and 4—**Table 1**). Concerning the average transformation efficiency (percentage of PCR-positive plants relative to the number of bombarded embryos), our protocol resulted in approximately 9.84% (± 2.49) efficiency rate, as described in **Table 1**. To guarantee a similar transformation efficiency as determined by the preliminary screening of T0 matrixes, we also analyzed the heritability of the transgene in T1-segregating plants. The average of heritability in T1 plants was 52.89% (± 11.23), demonstrating a similarly high ratio of T1 plants carrying out the transformation cassette as previously described for other soybean transformation methods (**Table 2**, Pareddy et al., 2020). In addition, as expected from an *Agrobacterium*-mediated transformation method, the positively recovered T1 plants harbor only a single copy or two copies of the transgene (**Table 3**). The copy number was estimated by qPCR analysis, according to Yi and Hong (2019). A standard DNA curve (1–10^-5^ ng) was obtained using a binary plasmid carrying an endogenous reference gene and a transgene against the linear cycle threshold (Ct) values. The absolute amount of the endogenous gene and the transgene was calculated based on the standard curve, as follows: A_gene_ = S_gene_ x Ct + I_gene_, in which A = absolute amount; S = curve slope; I = intersection curve. The relative copy number was estimated by dividing the absolute amount of the transgene by the absolute amount of endogenous reference gene. Troubleshooting and limiting steps are described in **Table 4**.

**Table 1:** Regeneration efficiency (number of recovered seedlings/number of bombarded embryos x 100) and transformation efficiency (number of PCR-positive T0 plants/number of bombarded embryos x 100).

Series	Bombarded Embryos	Recovered Seedlings	Selective Marker Gene in Vector Backbone	Selective Agent		Regeneration (%)	PCR (+) Plants	Efficiency (%)
1	150	48	*ahas*	Imazapyr	32		21	14
2	150	40	*ahas*	Imazapyr	26.7		14	9.3
3	300	282	*bar*	-*	94		22	7.3
4	300	232	*bar*	-*	77.3		29	9.6
5	300	99	*ahas*	Imazapyr	33		27	9

These data resulted from five independent series of soybean embryo axis transformations.

(*) Ammonium glufosinate or any other topic herbicide, which does not accumulate into shoot apex cells, cannot be used in selective DRM to avoid radicle necrosis.

**Table 2:** Gene-insertion heritability in T1 transformed plants.

Series of selected T0-matrixes	Number of T0-selected matrixes	Assayed T1 plants	PCR (+)T1 Plants	Heritability*
1 and 2	7	37	20	54.05%
3 and 4	15	107	44	41.12%
5	10	75	47	63.51%

(*) The heritability rate (number of PCR-positive T1 plants x 100/number of assayed T1 plants) was calculated according to the number of PCR-positive T1 plants obtained from the putative T0-matrix selected on the first screening of transformed plants.

**Table 3:** Transgene copy number in T1 plants estimated by qPCR.

PCR (+) T1 assayed plants	Estimated transgene copy number	Number of Plants	(%)
24	1	18	75
	2	6	25
	3	0	–

**Table 4:** Troubleshooting and limiting steps of *Agrobacterium*-mediated soybean embryonic axis transformation.

Step	Problem	Expected Consequence	Possible Solution
**Medium Preparation*(Day 1)***	pH checking	The wrong pH can modify the ionic state of chemical components in culture media, interfering with their bioavailability during plant development.	Check the pH during medium preparation using a calibrated and accurate pH meter. Adjust the pH precisely.
	Hormones and other chemical supply	The absence or the inactivity of hormones in culture media does not allow the shoot and root induction during plant regeneration.	Do not forget to supply the medium with all hormones and other chemicals during its preparation. Follow the reagents setup and storage recommendations exactly. Check the medium temperature before the supplementation with hormones.
	Selective agent—ammonium glufosinate	The presence of ammonium glufosinate in DRM may rot the embryonic axes during the regenerative process.	If the selective marker gene confers resistance to ammonium glufosinate, prepare DRM without a selective agent, and after seedling regeneration, follow the recommendations to select putative transgenic plants.
**Tungsten-coated membrane preparation*(Day 1)***	Dried membrane	Depositing tungsten at the dried membrane surface does not allow its adhesion and correct spread, decreasing the number of particles bombarded against the shoot meristem and the wounding ratio for the bacterial infection.	Be aware of the membrane storage in ethanol and prepare the tungsten particles before removing it. Quickly apply the particles at the membrane surface.
**Embryonic axis excision and shoot exposing*(Day 2*)**	Damaged/broken embryos	Damaged shooting cells may not regenerate into an apical system, resulting in low regeneration frequency.	Remove the embryo axis carefully with the help of suitable tweezers and scalpel. Check the integrity of the shoot apex using a stereoscope. If embryo removal displays any resistance, make sure that the seeds were well hydrated. It is possible to hydrate them for more time.
**Bombardment preparation*(Day 2)***	Embryo position	Shooting damage at the death zone or low frequency of particle bombardment, resulting in low transformation efficiency.	Avoid the positioning of the embryo axis at the death zone, where the incidence of microparticles is high during the bombardment. Make sure that the embryos are oriented vertically and completely dried to guarantee maximum exposure of the shoot cells.
**Embryo axis *in vitro* cultivation *(Up to 3 weeks)***	Bacterial and fungal contamination	The availability of nutrients in culture medium enhances the chance of bacterium and fungus development during plant regeneration, culminating in unviable embryos that can result from many factors, including pathogen-mediated embryo oxidation. Biological contamination represents the most limiting factor, leading to low regeneration frequency and transformation efficiency.	Verify the quality of seeds. Sterilization must be well executed in a laminar flow chamber. During the regenerative process, be aware of any contamination, taking care to eliminate it by removal or suitable antibiotic treatment.

## Discussion

Soybean is one of the most important nutritional crops around the world and is considered the main source of oil and protein for animal and human food and feed (Han et al., 2016; Li et al., 2017). For this reason, soybean has become the largest commercial crop planted worldwide, and genetic breeding programs have been extensively developed to generate new soybean varieties adapted to different climates and other agribusiness demands. Soybean has attracted attention between the main targets of modern molecular breeding programs and was one of the earliest genetically modified crops to be introduced for commercial cultivation (Wang et al., 2006; Krishna et al., 2010; Yamada et al., 2012). However, the genetic transformation of soybean is not trivial, and the described protocols display very low efficiency. It is expected that one superior cultivar of soybean with reasonable value and agricultural importance is a result of thousands of transformation events (Wang et al., 2006). Fundamental science would also benefit tremendously from the development of a simple and efficient transformation method because many genes in the soybean genome are still annotated as unknown functional genes (Schmutz et al., 2010; Li et al., 2017).

Despite the availability of well-established methods to deliver a foreign gene to plant cells, from the choice of explant to the DNA-delivery method, there are intrinsic factors that limit the efficiency of soybean transformation protocols. These factors are related to *A. tumefaciens* virulence, multiple-copy integration, the limiting size of integrative cassettes in the biolistics method and, mainly, the multiple steps of explant *in vitro* cultivation with subsequent plant regeneration. To overcome these problematic steps, our protocol combined the main advantages of each pre-established system of soybean transformation, and we developed a new method with higher plant regeneration and efficiency ratios.

*Agrobacterium*-mediated transformation is widely employed to generate transgenic plants. The main limitations of this DNA-delivery method are associated with the effectiveness of infection and explant regeneration, resulting in long and inefficient protocols. The growth stage of the *Agrobacterium* culture, culture concentration, infection time, medium composition and the regeneration capacity of explants directly affect the transformation efficiency (Zhou et al., 1983; Hinchee et al., 1988; Bailey et al., 1993; Donaldson and Simmonds, 2000; Paz et al., 2006).

The infectivity of plant cells by *Agrobacterium* ssp. is dependent on chemotaxis. Throughout cell wounding, metabolite exudates are released by plant cells and perceived by bacteria that trigger the process of colonization and T-DNA delivery. During the logarithmic growth stage, the bacteria display increased metabolic effectiveness, resulting in enhanced infectivity (Zhou et al., 1983; Paz et al., 2006). For soybean cotyledonary nodes, Li et al. (2017) described 96% efficiency of infection, which was monitored and reported by the highest GUS activity after the co-cultivation step of embryos and with a bacterial culture displaying an OD_650_ = 0.6. At this OD, the concentration of bacteria during the infection step is not too low to decrease its infection capacity and not too high to hamper its removal from explants before *in vitro* cultivation.

Many protocols of soybean transformation employ cotyledonary nodes isolated from germinated seeds as explants. After isolation, they are mechanically wounded, and submitted to the infection step. Reproductive plants are regenerated from the pluripotent cells present in this part of the embryo through multiple steps of *in vitro* cultivation, including shoot induction and elongation followed by rooting. The choice of cotyledonary nodes facilitates the generation of explants, as they can be easily isolated from germinated seeds. However, their efficiency of regeneration is very low, and shoot induction is considered the limiting step of this protocol, whose efficiency fluctuates between 2.5% and 4.0% (Paz et al., 2006; Song et al., 2013).

The problem of explant regeneration is partially overcome by medium supplementation with plant hormones (Gonbad et al., 2014; Li et al., 2017). The supplementation of SIM (shoot induction medium), SEM and RM with auxin, cytokines, and gibberellin analogs improves plant regeneration, raising the elongation efficiency to 26%–34%, although multiple steps of tissue culture are still necessary (Li et al., 2017). However, the demand for successive medium changes leads to yield losses by contamination caused by excessive explant manipulation, which makes the protocol long and expensive and eliminates the effects of improved regeneration protocols on plant transformation efficiency.

In our *Agrobacterium-*mediated transformation protocol, we overcame problems of both plant regeneration and excessive manipulation by changing the explant type. We developed a method that takes advantage of *Agrobacterium* ssp. T-DNA insertion and explored the high regenerative capacity of shoot cells in the embryonic axis, also derived from mature seeds, already reported by biolistic protocols (Rech et al., 2008). In our protocol, the embryo axis displayed 27% to 94% regenerative capacity in one-step *in vitro* tissue culture after co-cultivation. In the presence of the selective agent imazapyr, the average plant regeneration was 30.56 (± 2.76%). Other protocols reported regeneration rates between approximately 11% and 34%, reached by medium supplementation with a diverse combination of growth hormones and antioxidants (Li et al., 2017; Pareddy et al., 2020), against DRM only supplemented with BAP. In the absence of a selective agent, the maximum regeneration rate was 77% to 94%. This rate was already expected because all bombarded shoot cells in the embryo axis display full capacity to develop into a new plant, reinforcing the high regenerative capacity of the soybean embryo axis that supported our explant choice.

Complete plant regeneration was performed only in the DRM, which was composed of basic nutrients of plant media and only supplemented with the cytokinin BAP and charcoal. BAP is necessary to activate shoot elongation after its induction in CCM, also supplemented with GA_3_ and charcoal, which stimulates natural geotropic mechanisms of rooting. The use of the embryonic axis offers an enormous advantage to the plant regenerative process and explant manipulation. The one-step regeneration in DRM comprises the main advantage of our protocol: it dismisses the continuous media-changes supplemented with several phytohormones and growth regulators demanded by *in vitro* tissue culture and introduce an efficient alternative to the long steps of tissue culture reported by other protocols with close-related or higher efficiency (Paz et al., 2006; Li et al., 2017; Pareddy et al., 2020).

Our results demonstrate high reproducibility regarding both regeneration and transformation efficiency, reaching an average of 9.84%. This value is close to those reported by other *Agrobacterium*-mediated soybean transformation protocols, which fluctuate between 2% and 10%, depending on the soybean genotype (Meurer et al., 1998; Olhoft and Somers, 2001; Liu et al., 2004; Li et al., 2017). It is also close to the frequency of transformation obtained by biolistic protocols (Rech et al., 2008). In addition, our protocol offers a stable genetic transformation workflow, with a heritability of almost 50% in T1-segregating plants, reinforcing its usability and efficiency. As desirable by opting for the *Agrobacterium-*mediated method of plant transformation, the transgenic events harbor few T-DNA insertions, as 75% of the recovered germlines harbor a single transgene insertion and 25%, double insertions. Finally, the described protocol, which combines biolistic delivery and *Agrobacterium*-mediated transformation, provides a feasible, highly reproductive and efficient soybean transformation method.

## Data Availability Statement

All datasets presented in this study are included in the article/supplementary material.

## Author Contributions

BM, IL-T, and CM designed the study, performed protocol adjustment and supervised all the experiments. BM, IL-T, NS, LP, and CJ performed plant transformation series. MG-D-S was the leading researcher for all the work and provided intellectual input and financial support. MS and LM provided material and technicians resources. EF provided intellectual input and critically read and corrected the manuscript. BM, IL-T, and CM wrote and amended the manuscript.

## Conflict of Interest

The authors declare that the research was conducted in the absence of any commercial or financial relationships that could be construed as a potential conflict of interest.
